# Assessing the Impact of Chemical Algae Management Strategies on Anurans and Aquatic Communities

**DOI:** 10.1002/etc.5514

**Published:** 2022-12-09

**Authors:** Courtney Dvorsky, Kambrie Riddle, Michelle Boone

**Affiliations:** ^1^ Department of Biology Miami University Oxford Ohio USA

**Keywords:** Aquashade, copper, amphibians, algae, management

## Abstract

Pond management with chemical and biological agents that reduce overgrowth of algae is an important means of maintaining water quality in residential ponds, yet the effects on nontarget species are not fully understood. We assessed the impact of Aquashade (a common nontoxic pond dye) and copper sulfate (a toxic algaecide) on American toad (*Anaxyrus americanus*), northern leopard frog (*Lithobates pipiens*), and Cope's gray treefrog (*Hyla chrysoscelis*) metamorphosis in outdoor mesocosm experiments. We also evaluated the relative impact of tadpole grazing versus chemical treatment on phytoplankton and periphyton abundance. We found no significant effects of pond management treatment on anuran metamorphosis, suggesting that addition of Aquashade and copper sulfate at tested concentrations does not significantly impact anurans under these experimental conditions. Interestingly, we found that the presence of tadpoles more strongly reduced algal abundance than Aquashade or copper sulfate by significantly decreasing phytoplankton and periphyton abundance over time. The present study suggests that anuran tadpoles may be effective at maintaining water quality, and that Aquashade and copper sulfate may have minimal effects on amphibian metamorphosis. *Environ Toxicol Chem* 2023;42:213–224. © 2022 The Authors. *Environmental Toxicology and Chemistry* published by Wiley Periodicals LLC on behalf of SETAC.

## INTRODUCTION

Loss of habitat poses a major threat to aquatic species (Hamer & McDonnell, 2008). However, creation of ponds has been increasing in residential areas, potentially restoring habitat for native biodiversity (Renwick et al., 2005; Smith et al., 2002), particularly in areas of low‐density exurban, residential development (Davis et al., 2021; Ewers & Didham, 2006; McKee et al., 2003; Theobald, 2004). The management of created pond habitat can vary drastically based on individual landowner preferences in aesthetics, recreational use, and financial investment (Blaine et al., 2012; Hansen et al., 2005; Urban & Roehm, 2018). Many landowners use chemicals as a tool to decrease aquatic vegetation (Blaine et al., 2012; Carey et al., 2013; Loman & Lardner, 2006; Martini et al., 2015; Peltzer et al., 2008) and in a survey of exurban landowners, 46% reported adding pond dye to their pond to improve aesthetics and control aquatic algae (Davis et al., 2021). Given the increase in exurban development on the landscape (Brown et al., 2005; Robinson, 2012), and consequent pond creation, it is increasingly important to evaluate the impact of pond management practices on native species (Locke et al., 2019).

Aquashade and copper sulfate are two common chemicals used to manage residential ponds with aquatic plant overgrowth (Lynch, 2009). Copper sulfate has historically been used to decrease algal growth, although it is known to be toxic to other aquatic organisms (García‐Muñoz et al., 2009; Lynch, 2009; US Environmental Protection Agency [USEPA], 2013). Copper sulfate varies in its ability to be effective because its toxicity depends on several factors, including water alkalinity, pH, temperature, and water body depth (Button et al., 1977; Chiari et al., 2015; Kaplan & Yoh, 1961; Murray‐Gulde et al., 2002; Straus & Tucker, 1993). Applied to an entire lake ecosystem (at 0.1 ppm), Effler et al. (1980) observed a decrease in phytoplankton biomass and bacteria for several days following copper sulfate application; however, after less than a week both had returned to levels observed prior to copper sulfate application. No impact was observed on zooplankton biomass (Effler et al., 1980). The present study suggests that initial application of copper sulfate can stress aquatic ecosystems but decreases in effectiveness over time if not reapplied. In laboratory studies, northern leopard frog (*Rana pipiens*) tadpoles exposed to copper sulfate concentrations >0.15 ppm showed increased mortality (Chen et al., 2007; Lande & Guttman, 1973). In addition, García‐Muñoz et al. (2009) found Natterjack toad (*Epidalea calamita*) tadpoles exposed to >0.20 ppm copper sulfate had significantly decreased growth compared with controls, but the timing of copper sulfate application mattered, such that exposure at embryonic life stages had more negative impacts compared with tadpole life stages. Other tadpole species have shown similar responses, with those exposed to copper sulfate early in development experiencing increased time to metamorphosis and exhibiting significant physiological impairments such as edema, resulting in high mortality (Carvalho & Fernandes, 2006; Chen et al., 2007; Garcia‐Munoz et al., 2010).

Aquashade is marketed as a nontoxic pond dye (composed of tartrazine and erioglaucine) that directly blocks light and indirectly controls aquatic plant growth (Suski et al., 2018; USEPA, 2005). When applied to an entire lake ecosystem at 1.5 ppm, Aquashade decreased aquatic photic depth by 50%, phytoplankton biomass by 60%, and zooplankton biomass by 55% (Batt et al., 2015). In contrast, Aquashade applied at the recommended dose of 2.0 ppm in a 600‐L mesocosm experiment resulted in no change in phytoplankton biomass and an increase in zooplankton abundance; however, species composition became less diverse with Aquashade exposure (Suski et al., 2018). Ludwig et al. (2010) found Aquashade applied at twice the recommended dose (4.0 ppm) had no direct impact on fish fingerling abundance, weight, or survival in several species. Although studies focused on impacts to nontarget aquatic organisms are lacking, Bartson et al. (2018) found a pond dye similar to Aquashade (Tetra Pond Water Shade) had no impact on southern leopard frog (*Lithobates sphenocephalus*) tadpole response to predator cues (Western mosquitofish) or tadpole behavior when applied up to twice the recommended dose. Given the variability in these experimental results in the ability of Aquashade to decrease algal growth and impact on nontarget organisms, further investigation is necessary to elucidate the impact of Aquashade use in aquatic ecosystems.

Phytoplankton and periphyton are essential in bottom‐up aquatic ecosystem processes, therefore it is important to understand how various management strategies impact aquatic resources and communities (Mallory & Richardson, 2005; Rowland et al., 2017). Although Aquashade and copper sulfate are examples of common chemical management treatments used to decrease aquatic plant growth, they can impact larval anurans by limiting their primary food resource (Boone & James, 2003; Loman, 2001; Relyea, 2005). Native biological control, via tadpole grazing, offers another option for controlling overproduction of algae. Because tadpoles graze on both phytoplankton and periphyton (Dickman, 1968; Loman, 2001; Pryor, 2003), creation of pond habitat that attracts anurans could provide a natural form of bioremediation (Rowland et al., 2017).

We conducted two experiments to determine the effects of algal management using Aquashade and copper sulfate on anuran metamorphosis. Furthermore, we compared the addition of tadpoles, Aquashade, or copper sulfate to experimental mesocosms to evaluate their impact on phytoplankton and periphyton abundance. We hypothesized that Aquashade and copper sulfate would negatively impact anurans directly through mortality and reduced growth, and indirectly through decreased algal food resources. We also hypothesized that tadpole grazing would directly reduce algal food resources.

## MATERIALS AND METHODS

### Anuran egg collection

We collected nine partial northern leopard frog (*R. pipiens*) egg masses from a vernal pool at Talawanda High School in Oxford, Ohio, USA on March 21 and 22, 2017, seven partial American toad (*Anaxyrus americanus*) egg strings from Rush Run Wildlife Area in Somerville, Ohio on April 6, 2017, and six partial Cope's gray treefrog (*Hyla chrysoscelis*) egg masses from six breeding pairs at Miami University's Ecology Research Center (ERC) in Oxford, Ohio on May 27 and 28, 2017. In 2019, we conducted another study with four partial egg masses of Cope's gray treefrogs from four breeding pairs on May 27, 28, and 29, 2019 collected from the ERC. We placed eggs in containers with natal pond water. The containers with eggs were then brought back to the laboratory and placed in an environmental chamber at 23 °C, where they were monitored daily for hatching. Hatched tadpoles from all clutches were mixed and placed in new containers of dechlorinated water and monitored daily until they reached free swimming stage, Gosner Stage 25 (Gosner, 1960). We changed the water in tadpole containers daily and fed tadpoles TetraMin fish flakes until they were added to outdoor mesocosms.

### Mesocosm set‐up

**Figure 1:**
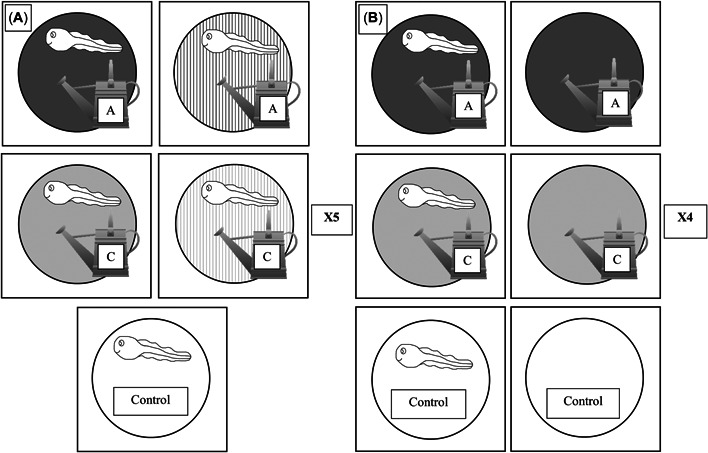
The experimental design for 2017 (**A**) and 2019 (**B**). In 2017 there were five treatments: two pond management treatments (Aquashade [A] and copper sulfate [C]) and two doses (high and low), with one control with no treatment; each treatment had five mesocosm replicates. In 2019, there were six total treatments: two pond management treatments (Aquashade and copper sulfate), two tadpole treatments (present or absent), and two control treatments; each treatment had four mesocosm replicates.

In 2017, we set up two separate experiments with 25 mesocosms on March 23, 2017 for spring breeding species (American toads and northern leopard frogs) and 25 mesocosms on May 23, 2017 for gray treefrogs (1.83 m in diameter, 1,480 L volume; Figure 1A). On March 24, 2017 and May 24, 2017, we filled polyethylene cattle tank mesocosms with 1000 L of Oxford city water, and on March 25, 2017 and May 25, 2017 added 1 kg of leaf litter (from a mixed deciduous forest with oak, beech, and maple) collected from Miami University's Natural Areas. We inoculated mesocosms with zooplankton and algae from a source pond located at the ERC on March 26, 28, and 30, 2017, and May 26, 28, and 30, 2017. Tadpoles were added to mesocosms once they had reached the free‐swimming stage, Gosner Stage 25 (Gosner, 1960). We added 30 northern leopard frog tadpoles on April 1, 2017 and 30 American toad tadpoles on April 12, 2017 (experimental day 0); the spring breeding anurans were reared together in mesocosms. We added 30 Cope's gray treefrog tadpoles to mesocosms on June 10, 2017. All mesocosms were covered with a screen lid (2 × 2 mm) to prevent nontarget species from entering the artificial community.

In 2019, we set up a total of 24 aquatic communities on May 27, 2019 in mesocosms (Figure 1B) following the same methodology as in 2017. We added water inoculated with zooplankton and algae from a source pond located at the ERC to each mesocosm on May 26, 28, and 30, 2019. On June 7, 2019 (experimental day 0) we added 30 Cope's gray treefrog tadpoles to the 12 mesocosms randomly assigned to the tadpole treatments. All mesocosms were covered with a screen lid (2 × 2 mm) to prevent nontarget species from entering the artificial community.

### Experimental treatments

In 2017, we added Aquashade and copper sulfate (“pond management treatments”) at two concentrations (high and low). The high‐concentration treatment was the manufacturer recommended dose of each chemical, calculated to the volume of our mesocosms (1000 L), and the low‐concentration treatment was half of the calculated recommended dose. Experimental treatments included control, low Aquashade (0.95 ppm [mg/L]), high Aquashade (1.90 ppm [mg/L]), low copper sulfate (0.0392 ppm [mg/L]), and high copper sulfate 0.0196 ppm [mg/L]), five replicates in each case. We applied each treatment by adding the measured amount of each chemical to a watering can filled with city tap water and distributed each evenly across the mesocosm; the watering can was rinsed clean in between each treatment application. Pond management treatments were applied to mesocosms 1 week after tadpole addition (April 19, 2017 and June 19, 2017, respectively) to allow for tadpole acclimation. One‐hour post dosing, we collected a composite sample across replicates by collecting a 1‐L sample of water from each cardinal direction in each mesocosm replicate, from which a 50‐ml sample for each treatment was sent to Mississippi State University (Mississippi, USA) for chemical analysis to confirm our treatment dose. Results from chemical analysis from the initial experiment showed no detection of copper sulfate or tartrazine (a yellow dye in Aquashade); however, erioglaucine (a blue dye in Aquashade) was detected at 4.82 ppm; failure to detect chemicals may have been a result of small water sample.

We added Aquashade and copper sulfate on June 11, 2019, at the manufacturer recommended concentration (1.90 ppm Aquashade and 0.0392 ppm copper sulfate), following the methods used in 2017. The experimental treatments were control, only tadpoles present, Aquashade only, copper sulfate only, Aquashade with tadpoles, and copper sulfate and tadpoles, and four replicates per treatment totaling 24 mesocosms (Figure 1B). Twenty‐four hours after pond management treatment addition, we collected a 1‐L composite sample (instead of 50 ml) of each treatment (following methods in 2017) and sent it to Mississippi State University for chemical analysis. We collected a larger water sample for chemical analysis in this experiment to have a higher likelihood of detecting our treatment chemicals because they were at a relatively low concentration. The results from this chemical analysis showed copper sulfate detection at 0.031 ppm, erioglaucine detected at 0.392 ppm, and tartrazine was not detected. We collected another composite sample 1 week after initial treatment addition to determine if any degradation had occurred. The results from those samples sent to Mississippi State University detected 0.020 ppm of copper sulfate and 0.210 ppm of erioglaucine, indicating a 30% degradation over 7 days; tartrazine was not detected.

### Anuran metamorphosis endpoints

We monitored mesocosms daily for metamorphs (presence of at least one front limb, Gosner Stage 42; Gosner, 1960). We brought all metamorphs to the laboratory and placed them individually in small (6 × 6 × 3 in) plastic containers (lids had four small air holes) filled a third of the way with tap water. Each container was tilted on a shelf in the environmental chamber to allow the individual to have access to space with and without water. Containers were checked daily for tail absorption. Once their tails were fully resorbed (Gosner Stage 46) we recorded time to metamorphosis and mass to the nearest 0.001 g to determine mass at metamorphosis; in addition, survival and time to metamorphosis were determined. All metamorphosed individuals were euthanized in 1% buffered tricaine methanesulphonate. We terminated the mesocosm experiment on July 25, 2017 (experimental day 116) and August 7, 2017 (experimental day 57). We drained all mesocosms using a mesh filter and searched through the leaf litter for remaining tadpoles and found no remaining tadpoles in the mesocosms. In 2019, the experiment was terminated on August 1, 2019 (experimental day 56), after one metamorph had been removed over a 10‐day period.

### Aquatic endpoints analysis

In addition to anuran responses, we measured light availability, zooplankton abundance, and phytoplankton and periphyton abundance to determine the indirect effects of our pond management treatments on the aquatic communities. Throughout both experiments we measured light intensity weekly, using a LI‐COR photosynthetically active radiation sensor, LI‐190R, at the center of each mesocosm at three depths (above the surface of the water, directly under the surface of the water [7 cm], and at the bottom of each mesocosm [37 cm]) to determine the impact of darkening due to pond management treatment. To not alter light data, light measurements were taken in sunny conditions with minimal clouds, generally between 10:00 and 13:00. In addition, in 2019, we measured total zooplankton abundance twice during the experiment: 24 h after chemical dosing and 1 week prior to experimental shut down. Using a polyvinyl chloride water sampling device, we collected water samples from all four cardinal directions of each mesocosm and put them in a 5‐gallon bucket, which we then took a 1‐L composite sample from. Each sample was filtered using a zooplankton concentration net and preserved with 80% ethanol in a 40‐ml glass scintillation vial.

We also measured periphyton abundance by deploying two pool noodles (unanchored) with glass microscope slides inserted vertically in each mesocosm (experimental day 7); samples were collected weekly (experimental days 29, 36, 44, and 50). We collected two microscope slides and scraped them (15.24 $cm^{2}$ each slide, 30.48 $cm^{2}$ total area) using a razorblade to remove periphyton onto a Merck Millipore 0.7‐mm pore diameter fiber glass filter and put it in a glass scintillation vial with 15 ml of 80% buffered acetone. We measured phytoplankton abundance weekly (experimental days 29, 36, 44, and 50) by collecting a 100‐ml composite sample (1‐L samples were taken from each of the four cardinal directions in each mesocosm, and a 100‐ml sample was taken from that) from each mesocosm. We filtered all samples onto a fiberglass filter and put them in individual 40‐ml glass scintillation vials with 15 ml of 80% buffered acetone. All samples were stored in a refrigerator at 4 °C for 24 h and analyzed for chlorophyll *a* using a Turner 10 AU fluorometer. Phytoplankton and periphyton samples out of range were diluted by adding 1 ml of sample to 15 ml of 80% buffered acetone; the resulting chlorophyll *a* value was multiplied by a factor of 16.

### Statistical analysis

All statistical analyses were conducted in R Ver 3.5.1 (R‐Studio Team, 2021). Mesocosm was the experimental unit, and all anuran species were analyzed separately for each year. We conducted an analysis of variance (ANOVA) to determine differences in mass at metamorphosis. We analyzed differences in time to metamorphosis for each anuran species among treatments using a generalized linear model (GLM) with a poisson distribution (R package lme4), and differences in survival (metamorphs alive at end of the mesocosm experiment) across treatments using a GLM with a binomial distribution (R package lme4). We conducted a repeated‐measures ANOVA (R package stats) to determine differences in light intensity over time across treatments, and phytoplankton and periphyton abundance over time by pond management treatment and tadpole presence. Chlorophyll *a* abundance was log transformed to meet the assumption of normality. Furthermore, we analyzed differences in total zooplankton abundance over time by pond management treatment with a repeated‐measures ANOVA (R package stats). Total zooplankton abundance was rank transformed to meet the assumption of normality. We conducted a Dunnett's multiple comparison test (R package multcomp) as a post hoc analysis on all ANOVAs.

## RESULTS

**Figure 2:**
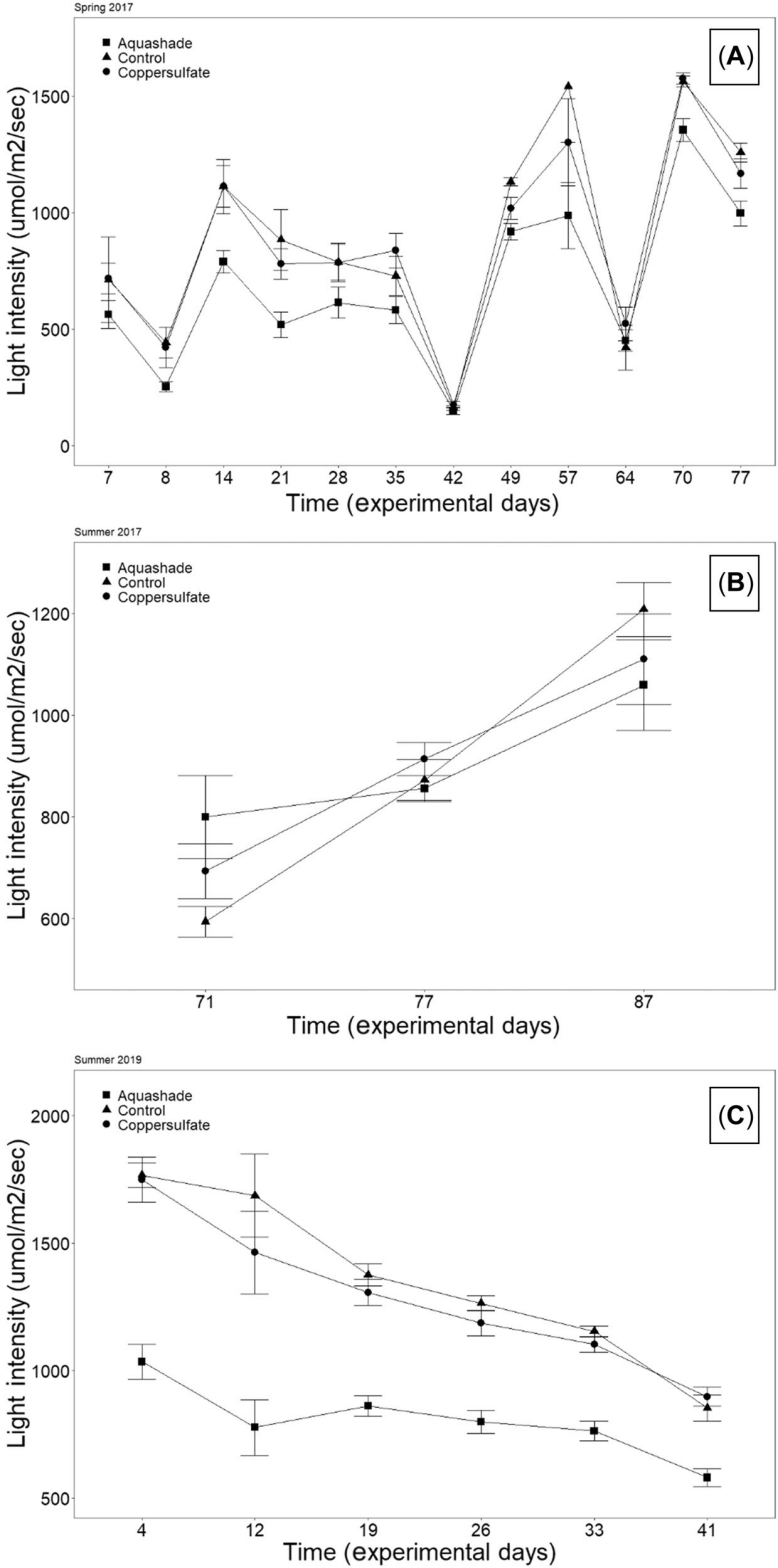
Average light intensity at the bottom of the mesocosm (0.37 cm) by pond management treatments. Mesocosms set up during spring breeders 2017 (**A**), summer 2017 mesocosms (**B**), and 2019 mesocosms (**C**). Plotted values are means + standard error. Significant differences were observed between Aquashade and control treatments, with Aquashade being significantly decreased.

**Figure 3:**
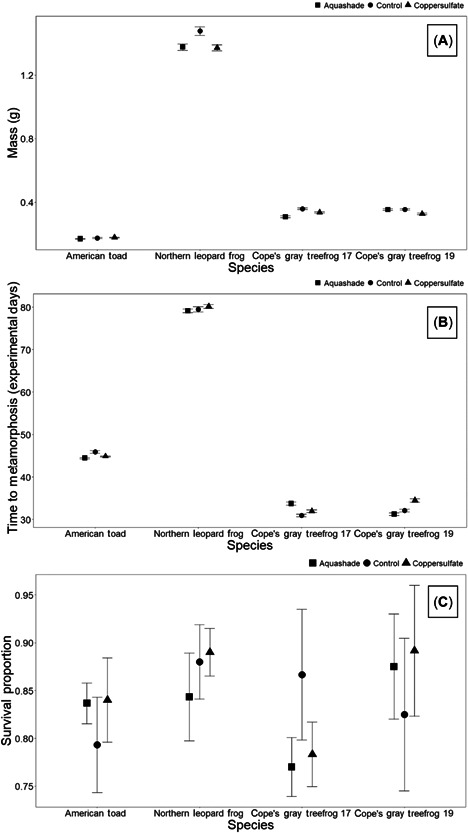
Anuran endpoints for both experiments: average mass at metamorphosis (**A**), average time to metamorphosis (**B**), and proportion of survival (**C**). Plotted values are means + standard error. No significant differences were observed in any anuran endpoint regardless of species, pond management treatment or year.

**Figure 4:**
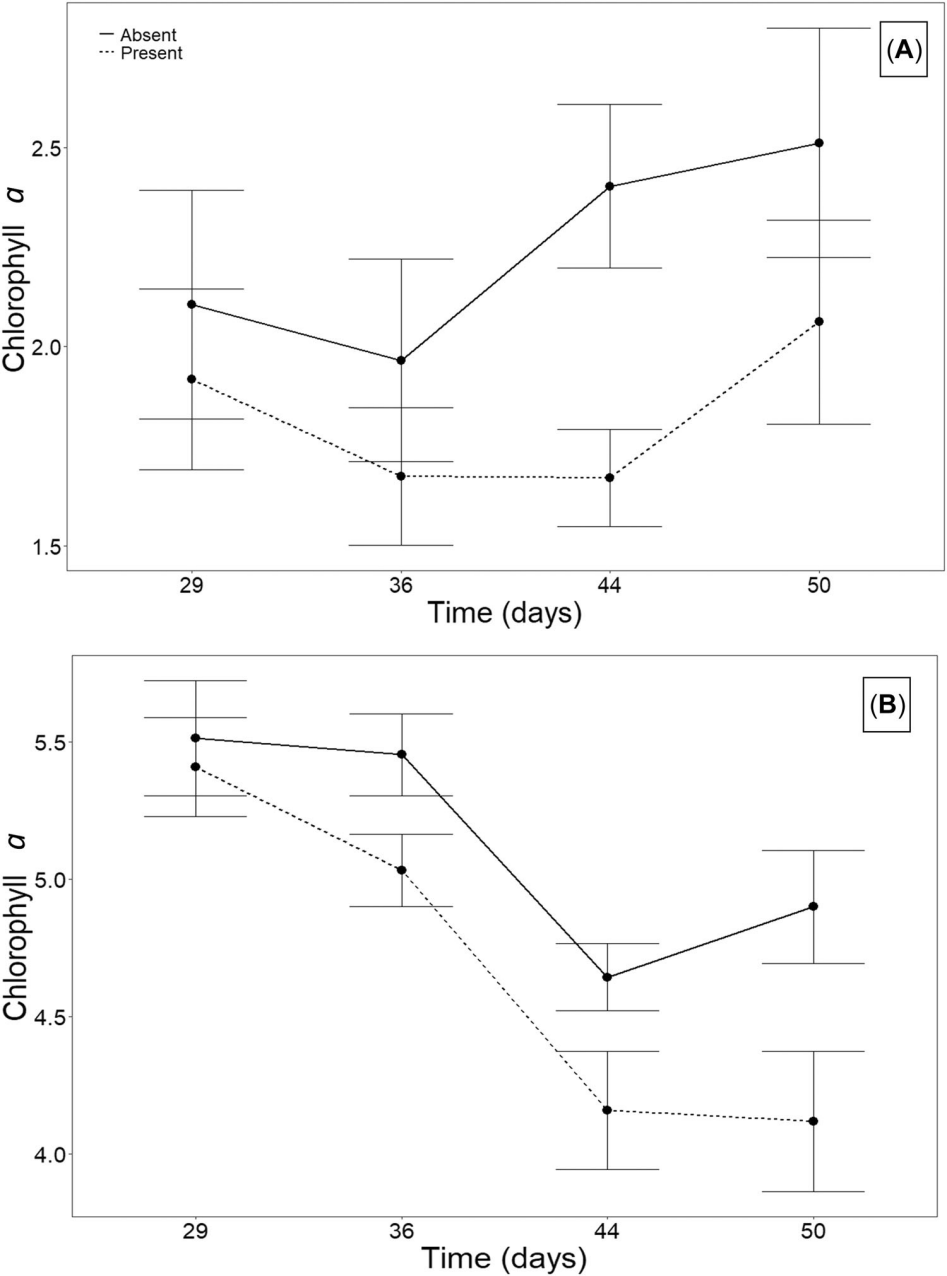
Average phytoplankton (**A**) and periphyton abundance (**B**) with tadpole presence over time (experimental days). Plotted values are means + standard error. Tadpole presence significantly decreased periphyton and phytoplankton abundance over time.

In 2017 and 2019, light intensity was significantly decreased in Aquashade‐treated mesocosms compared with control ponds at both low and high depths, regardless of Aquashade concentration (Table 1 and Figure 2). In 2019, the amount of light above mesocosms significantly differed among treatments; these differences are likely due to human error, with measurements potentially including some cloud cover contributing to data variation. Overall, we found no impact of pond chemical management on anuran mass at metamorphosis (Table 2 and Figure 3A), time to metamorphosis (Table 3 and Figure 3B), or survival (Table 2 and Figure 3C) for any anuran species in 2017 or 2019. In the 2019 study, gray treefrog tadpole presence was associated with a significant decrease in the abundance of both periphyton and phytoplankton chlorophyll *a* (Table 4 and Figure 4); in contrast, pond chemical management had no impact on periphyton or phytoplankton chlorophyll *a* abundance. In 2019, total zooplankton abundance significantly decreased over time; however, there were no significant differences due to chemical management or tadpole presence (Table 5).

**Table 1:** Summary of a repeated measures analysis of variance for light intensity at varying depths in mesocosms in 2017

Year	Response variable	Source of variation	*df*	*F* value	*p* value
2017	Average light above mesocosms	**Between subjects**			
		Management	4	1.023	0.419
		**Within subjects**			
		Date	11	66.154	**<0.001**
		Management × date	44	0.884	0.680
	Average light at 7 cm	**Between subjects**			
		Management	4	3.329	**0.030**
		**Within subjects**			
		Date	11	74.035	**<0.001**
		Management × date	44	0.779	0.837
	Average light at 37 cm	**Between subjects**			
		Management	4	7.204	**0.001**
		**Within subjects**			
		Date	11	68.127	**<0.001**
		Management × date	44	0.978	0.517
2019	Average light above mesocosms	**Between subjects**			
		Management	2	4.469	**0.025**
		Tadpole	1	0.071	0.792
		**Within subjects**			
		Date	5	14.421	**<0.001**
		Management × date	10	2.433	**0.012**
		Tadpoles × date	5	0.135	0.984
	Average light at 7 cm	**Between subjects**			
		Management	2	16.10	**<0.001**
		Tadpole	1	1.163	0.294
		**Within subjects**			
		Date	5	46.489	**<0.001**
		Management × date	10	1.370	0.205
		Tadpole × date	5	0.832	0.530
	Average light at 37 cm	**Between subjects**			
		Management	2	39.521	**<0.001**
		Tadpole	1	0.643	0.432
		**Within subjects**			
		Date	5	47.177	**<0.001**
		Management × date	10	3.761	**<0.001**
		Tadpole × date	5	0.130	0.985

Significant effects (*α* ≤ 0.05) are in bold text.

**Table 2:** Summary of analysis of variance for mass at metamorphosis (g) and survival for all species by management treatment in 2017 and 2019

Species	Year	Response variable	*F* statistic	*df*	*p* value
American toad	2017	Mass	0.332	4, 20	0.853
		Survival	0.248	4, 20	0.907
Northern leopard frog	2017	Mass	1.317	4, 20	0.298
		Survival	1.650	4, 20	0.201
Cope's gray treefrog	2017	Mass	0.843	4, 20	0.515
		Survival	0.588	4, 20	0.675
Cope's gray treefrog	2019	Mass	0.697	2, 9	0.523
		Survival	0.130	2, 9	0.880

**Table 3:** Summary of generalized linear model with a Poisson distribution on for time to metamorphosis (experimental days) by pond management treatment and dose (2017)

Species	Year	Response variable	*Z* value	*p* value	Coefficient
American toad	2017	Aquashade high dose	−0.418	0.676	−0.04 (0.09)
		Aquashade low dose	−0.356	0.722	−0.03 (0.09)
		Copper sulfate high dose	−0.349	0.727	−0.09 (0.09)
		Copper sulfate low dose	−0.258	0.797	−0.02 (0.09)
Northern leopard frog	2017	Aquashade high dose	−0.224	0.823	−0.0159 (0.07)
		Aquashade low dose	−0.059	0.953	−0.004 (0.07)
		Copper sulfate high dose	0.011	0.991	0.0008 (0.07)
		Copper sulfate low dose	0.129	0.897	0.009 (0.07)
Cope's gray treefrog	2017	Aquashade high dose	0.875	0.382	0.097 (0.11)
		Aquashade low dose	0.887	0.375	0.098 (0.11)
		Copper sulfate high dose	0.025	0.980	0.003 (0.11)
		Copper sulfate low dose	0.703	0.482	0.078 (0.11)
Cope's gray treefrog	2019	Aquashade	−0.135	0.893	−0.017 (0.126)
		Copper sulfate	0.651	0.515	0.080 (0.123)

**Table 4:** Summary of a repeated measures analysis of variance on periphyton and phytoplankton abundance (chlorophyll *a*) by pond management treatment, presence of tadpoles, date, and interactions

Response variable	Source of variation	*df*	*F* value	*p* value
Average periphyton chlorophyll *a*	**Between subjects**			
	Management	2	0.209	0.599
	Tadpoles	1	4.803	**0.003**
	Management × tadpoles	2	2.945	0.078
	**Within subjects**			
	Date	3	147.138	**<0.001**
	Management × date	6	3.254	0.776
	Tadpoles × date	3	0.652	0.383
	Management × tadpoles × Date	6	4.509	0.549
Average phytoplankton chlorophyll *a*	**Between subjects**			
	Management	2	0.747	0.483
	Tadpoles	1	4.173	0.057
	Management × tadpoles	2	0.602	0.559
	**Within subjects**			
	Date	3	2.037	0.120
	Management × date	6	1.096	0.377
	Tadpoles × date	3	0.921	0.437
	Management × tadpoles × date	6	3.142	**0.010**

Periphyton and phytoplankton chlorophyll *a* was log transformed to meet assumptions of normality. Significant effects (*α* ≤ 0.05) are in bold text.

**Table 5:** Summary of a repeated measures analysis of variance on zooplankton abundance by herbicide treatment, presence of tadpoles, date, and interactions

Response variable	Source of variation	*df*	*F* value	*p* value
Total number of zooplankton	**Between subjects**			
	Management	2	1.624	0.225
	Tadpoles	1	0.002	0.968
	Management × tadpoles	2	2.071	0.155
	**Within subjects**			
	Date	1	247.686	**<0.001**
	Management × date	2	2.152	0.145
	Tadpoles × date	1	3.452	0.080
	Management × tadpoles × date	2	4.780	**0.022**

Total zooplankton abundance data was rank transformed to meet assumptions of normality. Significant effects (*α* ≤ 0.05) are in bold text.

## DISCUSSION

Overall, the results from these experiments indicate that common chemical pond management strategies, when applied at recommended doses, have little impact on anuran metamorphosis, but also suggest that tadpoles could provide a natural ecosystem service via algal grazing at levels that exceed chemical management. Cope's gray treefrog tadpole presence resulted in a significant reduction in both periphyton and phytoplankton abundance, such that periphyton and phytoplankton abundance initially decreased when tadpoles were present and then increased after most individuals metamorphosed due to decreased grazing pressure. These results further support that both periphyton and phytoplankton are components of the gray treefrog tadpole's diet (Dickman, 1968; Loman, 2001; Morin & Johnson, 1988; Pryor, 2003), and that gray treefrog populations could control algal overgrowth during larval development. Morin and Johnson (1988) found similar patterns of periphyton and phytoplankton abundance over time due to variation in tadpole density and grazing pressure. Although tadpoles are generally not considered in aquatic algal management, creating quality habitats for amphibians could allow for a natural solution, with amphibians maintaining aquatic algal growth via natural grazing as an ecosystem service (Connelly et al., 2008; Holomuzki, 1998; Mendelson et al., 2006).

Although we did not observe decreased phytoplankton or periphyton due to chemical management treatment in our experiment, Hallingse and Phlips (1996) found the half maximal effective concentration (EC50) of Cutrine‐Plus (a common copper‐based aquatic plant management chemical) varies by algal species. It is possible in our experiment that the algal species inoculated in our mesocosms could have had an EC50 that was higher than the chemical treatments applied and therefore did not see any decreases in algal abundance. Furthermore, Murray‐Gulde et al. (2002) found that copper toxicity decreased with increasing algal cell density. Although we applied our copper sulfate treatment at the recommended dose, we still may not have had enough concentration based on initial algal density to directly decrease algal abundance and therefore observed stronger algal decrease from tadpole grazing. These studies emphasize the need to analyze algal species used in mesocosm experiments and take that into account when determining chemical treatment doses.

Aquashade is a pond dye used to decrease light availability in aquatic ecosystems as a mechanism to decrease algal growth (Tucker & Mischke, 2020). Aquashade's ability to decrease light availability was observed by Madsen et al. (1999), who applied Aquashade at 1 and 5 ppm, which decreased light availability by 10% and 30%, respectively, in a 3‐m water column. Spencer (1984) conducted an experiment on the concentration of Aquashade needed to decrease algal growth and determined application of at least 5 ppm was needed to decrease algal growth, but, similar to Madsen et al. (1999), found that concentrations from 1 to 3 ppm decreased light in a 1‐m water column by 50%. However, it should be noted that some species of algae, such as odor‐producing cyanobacterium (*Planktothrix perornata*), are able to grow in low‐light conditions and may not decrease with use of Aquashade because Aquashade is formulated to reduce water column light penetration at 600–650 nm (Tucker & Mischke, 2020). We did observe decreased light availability with Aquashade treatment in our mesocosms, however, we did not observe differences in algal abundance. Although we originally expected Aquashade to reduce phytoplankton and periphyton abundance and therefore indirectly impact aquatic grazers like tadpoles (Batt et al., 2015; Ludwig et al., 2010), we did not observe any negative impacts to anurans. This is encouraging because Aquashade is advertised as a nontoxic pond management chemical, and could be effectively used without harming nontarget organisms; however, it is important in future studies to determine the present algal species to be able to elucidate if the results observed are due to chemical concentration or algal species in mesocosms.

Prior studies have found evidence that copper sulfate is toxic to some anuran species (Lande & Guttman, 1973; McKim et al., 1978). García‐Muñoz et al. (2009) exposed natterjack toad (*Epidalea calamita*) embryos (Gosner Stage 3) and larvae (Gosner 19 and 25) to copper sulfate concentrations 0.05–0.40 ppm (mg/L), and determined that the LC50 at each Gosner stage was 0.22, 0.08, and 0.11 ppm, respectively. At all stages development was negatively impacted, growth was decreased by 50% when exposed to 0.20 ppm copper concentrations, and tadpoles had decreased escape behavior via shorter distance moved and abnormal displacement when exposed to predator cue. García‐Muñoz et al. (2010) similarly exposed five amphibian species (common toad [*Bufo bufo*], natterjack toad [*Epidalea calamita*], Spanish painted frog [*Discoglossus jeanneae*], Western spadefoot toad [*Pelobates cultripes*], and Perez's frog [*Pelophylax perezi*]) to copper sulfate at Gosner Stages 19 and 25 for 96‐h acute toxicity tests, and found growth reduction of 30% across all species and varied mortality, with common toad, natterjack toad, and Spanish painted frog having 90% mortality at 0.20 ppm, whereas Perez's frog experienced no mortality at the same copper concentration. Similar mortality of northern leopard frogs (*Lithobates pipiens*) was observed when exposed to 0.20 ppm of copper sulfate (Chen et al., 2007).

Christenson et al. (2014) used methodology similar to our experiment with application of Cutrine‐Plus (a copper‐based chemical) at two concentrations, the high concentration being the recommended dose (1.226 ppm) and the low dose half of the recommended dose (0.613 ppm), but observed significant wood frog larvae mortality at both concentrations. Although the experimental concentrations used in our experiment (high copper sulfate 0.0392 ppm [mg/L]) and low copper sulfate 0.0196 ppm [mg/L]) were at the recommended application dose or lower, the literature indicates negative effects at approximately 0.20 ppm, suggesting our copper sulfate concentrations were too low to observe negative effects on anurans. Moreover, although we did not observe differences of algaecide impact across species, observed variation in previous studies could be due to the timing of algaecide application and anuran emergence for the breeding season. Algaecide used earlier in the anuran breeding season could be more impactful due to less algal abundance, making it easier to control compared with later in the summer; however, more research would be necessary to elucidate the relationship between anuran emergence and algaecide application. Overall, the variation of algaecide impact across anuran species (García‐Muñoz et al., 2010) suggested the need to determine the LC50 of copper sulfate solution for each individual species before beginning the experiment.

Given the increase in residential development, consequent pond creation, and variation in pond management and chemical application, it is increasingly important to determine the impact of pond management on nontarget aquatic organisms. Our results indicate that at recommended or lower concentrations, Aquashade and copper sulfate had no negative impacts on anurans (American toad, northern leopard frog, and Cope's gray treefrog); however, our chemical treatments were at concentrations lower than in other studies, which could potentially explain why we did not observe negative impacts on anuran species. Given the increase in chemical use, with 46% of landowners reporting use of pond dyes to improve aesthetics and control aquatic algae (Davis et al., 2021), observing no negative impact on anuran species is encouraging for overall anuran species conservation, although the variation observed from other studies across chemical concentrations, application timing, and species studied supports the need for more research across a wide variety of environmental and biotic conditions. Although we did not observe impacts on algal abundance via chemical treatment, we did observe decreased algal abundance due to tadpole grazing, which suggests that creating ponds that are suitable for anuran species occupancy could provide natural bioremediation in local ponds (Urban & Roehm, 2018). Anurans are vital environmental health indicators in pond ecosystems (Mendelson et al., 2006) and natural anuran colonization could be a pond management strategy that benefits ecosystem health and maintains aquatic species in human‐dominated systems.

## Author Contributions Statement

**Courtney Dvorsky**: Conceptualization; Data curation; Formal analysis; Funding acquisition; Investigation; Methodology; Project administration; Writing—original draft; Writing—review & editing. **Kambrie Riddle**: Conceptualization; Data curation; Funding acquisition; Methodology; Project administration. **Michelle D. Boone**: Conceptualization; Funding acquisition; Methodology; Project administration; Supervision; Validation; Writing—review & editing.

## Data Availability

Data, associated metadata, and calculation tools are available from the corresponding author (dvorskc2@miamioh.edu), and are accessible through the data repository Figshare (https://figshare.com/account/home#/projects/151794).
